# Caregiver Challenges and Opportunities for Accessing Early Hearing Detection and Intervention: A Narrative Inquiry from South Africa

**DOI:** 10.3390/ijerph22040605

**Published:** 2025-04-11

**Authors:** Katijah Khoza-Shangase, Ntsako Precious Maluleke

**Affiliations:** Department of Audiology, School of Human and Community Development, Faculty of Humanities, University of the Witwatersrand, Johannesburg 2050, South Africa; precious.slp@gmail.com

**Keywords:** early hearing detection and intervention, deaf or hard of hearing (DHH), paediatric hearing loss, caregiver experiences, South Africa, family-centred care

## Abstract

Background: Early Hearing Detection and Intervention (EHDI) is essential for minimising the negative impact of childhood hearing loss on speech, language, and cognitive development. However, in low- and middle-income countries such as South Africa, various challenges hinder the implementation of EHDI services, leading to delayed diagnosis and intervention. Aim: This study explores caregivers’ experiences with EHDI services, identifying key challenges and facilitators affecting access and timely intervention. Methods: A narrative inquiry approach was used as part of a broader research initiative on family-centred EHDI. Nine caregivers of children who are deaf or hard of hearing (DHH) were purposively sampled, and data were collected through semi-structured interviews. Results: Thematic analysis revealed systemic and structural challenges, logistical and financial constraints, and caregiver-related factors that hindered access to EHDI services. Key facilitators included caregiver knowledge and advocacy, family support services such as counselling and South African Sign Language training, and high-quality audiological and educational services. Conclusions: Findings emphasise the need for policy-driven reforms, including expanding newborn hearing screening programmes, improving financial assistance mechanisms, and increasing public awareness. Addressing these challenges and leveraging facilitators can help South Africa align with global EHDI benchmarks and improve outcomes for DHH children.

## 1. Introduction

Hearing loss ranks as the fourth leading cause of disability worldwide, yet comprehensive data on its prevalence and epidemiology in low- and middle-income countries (LMICs), including South Africa, remains limited [1,2,3,4]. Despite global efforts to enhance early detection and intervention, there remains a significant gap in data collection and reporting, making it difficult to implement comprehensive strategies tailored to specific contexts. In South Africa, an estimated 6 out of every 1000 live births, or approximately 20 infants daily, are affected by congenital or early-onset hearing loss [5,6]. In this country and similar LMIC contexts, universal newborn hearing screening (UNHS) is not consistently implemented, and timely access to diagnostic and intervention services remains limited [2,5,6]. These numbers highlight the urgency for improved national screening programmes and increased healthcare infrastructure to accommodate the growing need for paediatric audiological care, particularly since Early Hearing Detection and Intervention (EHDI) is globally recommended as a strategy to mitigate long-term impacts of hearing loss. Given this reality, EHDI has become a critical objective within the audiology field to mitigate the well-documented communication, cognitive, and academic difficulties associated with delayed diagnosis and intervention [4,5,6]. Without timely identification and support, children who are deaf or hard of hearing (DHH) often face lifelong challenges that affect educational attainment, employment opportunities, and social inclusion.

UNHS serves as the foundational step in EHDI programmes and is standard practice in high-income countries (HICs), ensuring that hearing loss is identified as early as possible, thereby facilitating prompt intervention. However, South Africa has yet to achieve widespread implementation [7]. Research in this context highlights significant delays in the identification, diagnosis, and intervention for DHH children [8,9,10]. In LMICs like South Africa, EHDI implementation has been inconsistent and fragmented, with most public hospitals lacking UNHS [2,3,5,6,10,11]. Studies indicate that screening coverage is limited to specific provinces such as Gauteng and Western Cape, and even where screening is available, follow-up systems to ensure diagnostic testing and intervention are often weak [2,10,11]. For instance, children frequently receive diagnosis and amplification after the critical period for language acquisition, sometimes well after 12 months of age. Factors contributing to these delays include inadequate tracking systems, workforce shortages, limited parental awareness, and inconsistent availability of audiological and early intervention services [8,11,12,13,14]. While global guidelines propose screening by 1 month, diagnosis by 3 months, and intervention by 6 months, South Africa continues to face challenges and delays in achieving these benchmarks, especially in the public healthcare sector where the majority of children receive services. Additionally, access to EHDI services in South Africa remains deeply inequitable, shaped by socioeconomic disparities, geographic location, and resource allocation across provinces [2,5,6,8,9,10,11,12,14,15]. Children from rural or underserved areas often face significant delays in screening and diagnosis due to long distances to service points, a lack of trained personnel, and inadequate referral pathways. Moreover, financial constraints, linguistic barriers, and lack of awareness contribute to differential access and utilisation of early hearing services [14]. These inequities disproportionately affect families reliant on the public healthcare system, leaving many children at risk of missed or delayed identification of hearing loss and its consequences.

These delays have long-term consequences, including poorer speech and language development, lower academic performance, and reduced quality of life. These delays stem from multiple systemic challenges, including financial and human resource constraints, the concentration of audiologists within the private sector, high disease burdens, and logistical, organisational, and cultural obstacles [2,5,6,11,12]. Consequently, paediatric hearing loss is often deprioritized, receiving limited financial resources and minimal political commitment from the Department of Health [13,14]. The lack of prioritisation is further exacerbated by competing healthcare demands such as infectious diseases, maternal and child health concerns, and limited funding for non-communicable diseases, resulting in hearing healthcare receiving inadequate attention.

Facilitators to access to healthcare services, including EHDI, are not as evidently recorded in the literature, with the majority of studies focusing on challenges to implementation rather than solutions [15]. Identified facilitators include legislative support for EHDI, financial support, friendly audiologists, awareness of hearing loss, and availability of support services [8,15,16]. Positive provider–family interactions, including empathetic and culturally responsive communication by audiologists where “friendly audiologists” are described as those who are perceived as approachable, patient, and supportive—are characteristics that were especially valued in contexts where caregivers may feel disempowered or unfamiliar with technical processes. Legislation and policies that support universal screening and intervention frameworks are instrumental in ensuring systematic improvements in service delivery. The scarcity of reported facilitators, compared to challenges, may be due to limited research focus in this area and the lack of EHDI services in LMICs [15,17]. However, it is essential that the strengths and achievements of EHDI services be identified to ensure best practice and develop a standard of care, especially within the South African context [18]. By shifting focus towards identifying and amplifying facilitators, policymakers and practitioners can leverage existing strengths to enhance accessibility, efficiency and sustainability of EHDI services.

Ensuring adherence to EHDI principles is fundamental to the success of any effective intervention programme and is directly linked to improved developmental outcomes for DHH children [2]. In the South African context, guided by the Health Professions Council of South Africa (HPCSA) [19], these objectives include diagnosing hearing loss by no later than four months of age and initiating early intervention services by eight months. The HPCSA is the statutory body responsible for regulating health professions, including audiology, in the country. It provides guidance and oversight through professional boards, including the Professional Board for Speech, Language and Hearing Professions. The HPCSA’s EHDI guidelines are informed by international best practises but are adapted to accommodate the local context, including disparities in access to services, human resource limitations, and infrastructural constraints. These systemic challenges influence the feasibility of achieving the recommended timelines, particularly within resource-limited public sector settings. Achieving these targets set by the HPCSA requires the development and implementation of EHDI services that are contextually relevant and inclusive of caregivers and families as active participants in the intervention process. Family involvement has been widely recognised as a key determinant of positive intervention outcomes, as caregivers play a critical role in facilitating language development and communication access for children with hearing loss. Incorporating caregiver perspectives helps tailor EHDI services to be evidence-based, responsive to the local context, and aligned with the needs of children, caregivers, and families [2]. Additionally, integrating caregiver voices into policy and service design fosters a sense of ownership and ensures that interventions are both sustainable and practical within real-world settings. This study, therefore, aimed to explore caregivers’ experiences to identify challenges and facilitators in the EHDI process. By understanding these perspectives, this research seeks to inform future interventions that are both effective and contextually relevant, ultimately leading to improved outcomes for DHH children.

This study is part of a broader research initiative titled ‘Family-Centred EHDI: Caregivers’ Experiences and Evaluation of the Process and Practices in the South African Context,’ aimed at laying the foundation for a framework on family-centred early intervention (FCEI) for children who are DHH in South Africa. Although this research is rooted in the South African context, the narrative inquiry approach enables the identification of systemic, professional, and caregiver-related challenges that are also relevant to other LMICs and underserved communities worldwide. By capturing first-hand caregiver experiences, the study provides valuable insights into the lived realities of families navigating the complexities of paediatric hearing healthcare. The findings underscore universal themes in EHDI service delivery, including the critical role of family-centred care, the need for equitable access, and the importance of culturally responsive interventions, all of which have broader implications for global practice and policy development. Recognising these universal themes allows for cross-contextual learning, where solutions and best practices from one setting can inform strategies in others, contributing to a more inclusive and effective global approach to EHDI.

## 2. Materials and Methods

### 2.1. Design

This study employed a narrative inquiry approach [20] to gain a comprehensive understanding of caregivers’ lived experiences and identify actionable insights that could inform EHDI practices across diverse contexts. This methodology was chosen as part of a larger, ongoing research initiative on family-centred EHDI services in South Africa. The tool (interview guide) utilised in this study had been previously developed and validated as part of the larger study, ensuring consistency and alignment with the overarching research framework. Narrative inquiry was particularly suited for this research, as it allows for the exploration of deeply personal and context-specific experiences, providing a richer understanding of the challenges and facilitators caregivers encounter in navigating EHDI services. An individual narrative inquiry approach was selected over focus group methods to allow for a deeper, more nuanced exploration of each caregiver’s unique journey through the EHDI process. This approach enabled participants to reflect openly on their personal experiences, without the influence of group dynamics or social desirability pressures that can arise in focus groups. Given the sensitive and emotionally complex nature of early childhood hearing loss and the barriers to accessing care, individual interviews provided a safe and confidential space for caregivers to share intimate details about their challenges and decision-making processes. Additionally, the narrative approach aligns with the study’s intention to understand the temporal, contextual and individual factors influencing access, which may not emerge as richly in group settings.

### 2.2. Participants

Purposive, convenience sampling was employed for participant recruitment, enabling the researcher to select caregivers whose children were enrolled in early intervention preschools in Gauteng. Narrative inquiry typically requires a small number of participants to allow for an in-depth exploration of individual experiences, which is not feasible with a large sample size [21]. Participants were recruited telephonically until data saturation was reached, ensuring that no new insights emerged from additional interviews. The choice of participants was intentional to ensure representation of diverse socio-economic backgrounds and lived experiences. This variation provided a more comprehensive understanding of the challenges and facilitators impacting EHDI implementation in South Africa. Participants whose children had accessed EI services up to 10 years ago were included in the study. Importantly, many of these children were only enrolled in EI at later stages—some as late as six years of age—due to delays in identification and systemic gaps in EHDI implementation. Their inclusion allowed for a more comprehensive understanding of barriers and enablers across the continuum of care, particularly in the South African context where early access remains inconsistent. Moreover, retrospective accounts may offer unique insights, as caregivers are often better able to reflect on and articulate the full impact of their experiences over time.

Inclusion criteria:
  - Caregivers of a child who is Deaf or Hard of Hearing (DHH).
  - Caregivers of a child who was enrolled in an early intervention preschool in Gauteng between 2010 and 2018.
Exclusion criteria:
  - Caregivers of children with co-occurring conditions (such as cognitive or neurological impairments, or syndromic diagnoses) that could independently impact communication development, thereby introducing complexities beyond the study’s focus on hearing loss.
  - Caregivers who had limited or no interaction with South Africa’s EHDI services, including those whose children had not undergone formal audiological assessment or received intervention.
  - Individuals who were unable to participate meaningfully in interviews due to language differences or cognitive limitations that could not be accommodated within the study design.
  - Caregivers of children who had received all intervention services outside South Africa, where healthcare structures and access pathways differ significantly from the local context under investigation.

### 2.3. Data Collection

Data collection was conducted using a structured narrative interview schedule, outlined in Box 1. This interview schedule had been previously validated and utilised as part of the broader research initiative on family-centred EHDI services.

Box 1 **Interview script.**

- Tell me YOUR story.
- What were your expectations of the EHDI process?
- What do you think worked well or did not work so well with the EHDI process based on your experience?
- What made the EHDI process work well and what made it not work so well?

Source: Original

The narrative interview script was developed within a constructivist framework, informed by prior literature and the objectives of the overarching research study. A pilot study was conducted with two caregivers—whose children were also enrolled in early intervention preschools but who were not included in the main study—to assess the suitability of the interview script. Based on pilot study findings, minor refinements were made, including the rewording of two questions for improved clarity.

Originally, face-to-face interviews were planned as the primary data collection method. However, due to the COVID-19 pandemic and related restrictions [22], adjustments were made to include remote data collection methods. Three in-person interviews were conducted between January and February 2020, before the World Health Organization (WHO) declared COVID-19 a pandemic [23]. Subsequently, five additional interviews were conducted remotely, including two telephonic interviews and four videoconferencing interviews between March and June 2020. All interviews were audio-recorded with participant consent.

Interviews were conducted in English (5), Setswana (2), SeSotho (1) and IsiZulu (1), based on participant preference. The lead researcher, proficient in all these South African languages, conducted the interviews without the need for an interpreter. Each interview lasted an average of one hour and ten minutes. In addition to the narrative interview, participants completed a socio-demographic questionnaire, which gathered data on variables such as population group (race), home language, age, marital status, educational background, and economic status. This demographic data provided crucial contextual insights that complemented the narrative accounts and facilitated a deeper understanding of the lived experiences described by caregivers.

### 2.4. Data Analysis

All audio-recorded interviews were transcribed verbatim and anonymized using participant numbers and pseudonyms for children. Data were analysed using reflexive thematic analysis, following Braun and Clarke’s six-phase framework [24,25]:

1. Familiarisation with the data.
2. Generating initial codes.
3. Identifying themes.
4. Reviewing potential themes.
5. Defining and naming themes.
6. Producing the final report.

For interviews conducted in an African language, two bilingual translators translated the transcripts into English. To ensure accuracy, a third bilingual individual back-translated the transcriptions into the original language [26]. This back-translation process was undertaken to maintain data integrity and ensure that no meaning was lost during translation.

To mitigate potential bias and enhance the reliability of the findings, all researchers were actively involved in the data analysis process [26]. Reflexive thematic analysis emphasises the importance of researcher engagement with the data, ensuring thoughtful interpretation and multiple perspectives in the analytical process. The involvement of multiple researchers allowed for the exploration of diverse assumptions and facilitated richer interpretations of the meaning embedded within the data [27]. Regular debriefing sessions were conducted to discuss emerging themes, refine coding strategies, and ensure consensus among researchers, further strengthening the credibility of the findings.

The socio-demographic questionnaire data were analysed using descriptive statistics, providing an overview of participant characteristics and contextual factors relevant to the study.

### 2.5. Rigor and Trustworthiness

To ensure the rigor and trustworthiness of the study, several strategies were employed [28]. Credibility was enhanced through prolonged engagement with the data, investigator triangulation and member checking, where participants were given the opportunity to review and verify their transcripts. Transferability was ensured by providing a detailed description of the study context, methodology and participant demographics, allowing future researchers to assess applicability in similar settings. Dependability was addressed through a well-documented research process, ensuring that the study could be replicated. Confirmability was maintained by keeping an audit trail of decision-making processes, including reflexive journaling by the lead researcher to acknowledge potential biases and influences on data interpretation.

### 2.6. Ethical Considerations

Ethical approval for this study was obtained from the University of the Witwatersrand’s Human Research Ethics Committee (Medical) (Protocol Number: H19/06/16) prior to data collection. Informed consent was obtained from all participants, ensuring they were fully aware of the study objectives, procedures, potential risks and their right to withdraw at any stage without consequence. Confidentiality and anonymity were maintained by assigning pseudonyms and securely storing all data. Participants were also informed about the measures in place to protect their privacy, including encrypted storage of audio recordings and transcripts. Additionally, given the sensitive nature of discussing experiences related to their child’s hearing loss, participants were offered access to counselling support should they require emotional assistance following the interviews.

## 3. Results

### 3.1. Description of Participants

Participants’ socio-demographic profiles are summarised in Table 1 and Table 2. Fifteen caregivers were initially approached, and nine caregivers consented to participate, yielding a recruitment rate of 60%. All nine participants were female, with a mean age of 39 years (SD = 6.5). Five participants were Black, two were White, one was Multiracial, and one was Indian. The highest level of education attained varied, with one participant holding a postgraduate degree, three holding post-matric diplomas, three having completed matric (Grade 12), and two having Grade 11 or lower. Of the nine participants, five were employed, two were self-employed, and two were unemployed. Two participants had two children diagnosed with hearing loss, bringing the total number of children represented in the study to eleven.

The children’s demographic and audiological profile revealed that caregivers were responsible for seven male and four female children who were DHH. Their ages ranged from 6 to 15 years with a mean age of 10.2 years (SD = 1.1), with an age of diagnosis spanning from birth to 30 months (mean age: 12 months). All children had congenital or early-onset bilateral hearing loss and were fitted with hearing aids and/or cochlear implants bilaterally.

From the narrative interviews, themes emerged that captured the caregivers’ experiences. The themes are categorised into challenges and opportunities in accessing and navigating the EHDI process. This study identified three major challenges (systemic and structural challenges, logistical and financial constraints, and caregiver-related challenges) and three key facilitators (caregiver knowledge and advocacy, family support services, and positive healthcare and educational experiences) in the EHDI process.

### 3.2. Challenges to EHDI Access

The following are the three key themes with their subthemes depicting challenges to EHDI access within the South African context:

Theme 1: Systemic and Structural Challenges

Firstly, delayed diagnosis due to healthcare provider practices emerged as a subtheme. Six participants reported that healthcare workers (HCWs) dismissed their concerns regarding their child’s hearing loss, leading to a delayed diagnosis. Newborn hearing screening was not conducted for any of the children.

*‘When I asked the nurse why she was not talking at almost two years, she said she didn’t know.’*

(CG2)

Secondly, fragmented service delivery and multiple consultations emerged as another subtheme. Two participants described being referred to multiple HCWs before reaching an audiologist, prolonging the diagnostic process.

*‘We had to go to doctor, to doctor, to doctor. There were so many people.’*

(CG1)

Thirdly, mandatory use of English in EHDI services emerged as a systemic and structural challenge that caregivers confronted. Six non-English-speaking participants reported being advised to speak only English at home due to the unavailability of multilingual speech therapy and education services.

*‘Speech therapy was in English because at the time there were no Black speech therapists.’*

(CG3)

Theme 2: Logistical and Financial Challenges

Firstly, under this theme, challenges with accessibility of services emerged. Five participants reported that essential EHDI services were located far from their homes, requiring extensive travel. Three participants relied on public transport, making the journey even more difficult.

*‘It was quite a road to travel. I would drive 350 km a day to get him to talk.’*

(CG8)

Secondly, the financial burden of EHDI-related costs was also identified. Seven participants struggled with the prohibitive costs associated with hearing aids, therapy sessions, and related medical expenses. Some medical aids did not fully cover the cost of assistive devices.

*‘It really comes down to, are you financially able to give your child the support they need?’*

(CG5)

Thirdly, the impact on employment and daily life emerged as a logistical and financial challenge. One participant switched to permanent night shifts to accommodate her child’s numerous appointments.

*‘I had to change to night shift to keep up with appointments and minimize absenteeism.’*

(CG3)

Theme 3: Caregiver-Related Challenges

Firstly, limited awareness of paediatric hearing loss was one key caregiver-related challenge identified. Two participants reported not recognising the signs of hearing loss due to a lack of awareness about speech and language developmental milestones.

*‘If the crèche hadn’t picked it up, we wouldn’t have known something was wrong.’*

(CG1)

Secondly, limited paternal involvement in decision-making was another subtheme. Two caregivers reported that fathers were largely absent from appointments and decision-making regarding cochlear implantation and therapy.

*‘At that time, he really didn’t understand because he didn’t receive counselling.’*

(CG7)

### 3.3. Facilitators of EHDI Access

The following are the three key themes with their subthemes illustrating facilitators of EHDI access within the South African context:

Theme 1: Caregiver Knowledge and Advocacy

Self-education and proactive decision-making emerged as an important facilitator. Three caregivers reported using their acquired knowledge to advocate for early testing and intervention for other children in their communities.

*‘When Karabo was born, I asked them to do the tests immediately.’*

(CG3)

*‘I took my domestic worker’s child for tests, and it was determined that she was deaf.’*

(CG8)

Theme 2: Support Services for Families

Firstly, counselling and parent support networks were identified as important support services for families. Participants who accessed counselling services reported an easier transition in accepting their child’s hearing loss.

*‘Hi-Hopes came to visit us and helped us accept our child’s hearing loss.’*

(CG1)

Secondly, South African Sign Language (SASL) training for parents was the next subtheme that emerged. Two participants attended SASL training provided by their child’s school, which improved communication and academic support at home.

*‘The first year, we went for Sign Language classes every Saturday.’*

(CG1)

Thirdly, financial assistance for EHDI services was identified as another subtheme. Three participants received financial aid or sponsorships for their child’s medical and educational needs.

*‘Audiologist B got a sponsor to pay for Karabo’s preschool fees.’*

(CG3)

Theme 3: Positive Healthcare and Educational Experiences

Supportive and compassionate audiologists came up as a key subtheme. Caregivers appreciated audiologists who provided both technical support and emotional encouragement.

*‘I don’t see her as an audiologist; I see her as my sister or mother because she was always there for me.’*

(CG3)

Additionally, quality of specialised schools for DHH learners was another subtheme illustrating positive healthcare and educational experiences. Caregivers noted positive changes in their children’s progress after enrolling in specialised schools.

*‘At first, I was unsure about the school, but once my son was there, he was a different child.’*

(CG6)

## 4. Discussion

This study explored the experiences of caregivers navigating access to EHDI services for their children within the South African context. Through a narrative inquiry approach, it uncovered a range of challenges and opportunities caregivers encountered, including systemic barriers, socio-cultural influences, and service-level gaps. The findings highlight the complex interplay between healthcare accessibility, caregiver agency, and the responsiveness of existing EHDI systems. These narratives offer critical insights into how context-specific realities shape early intervention outcomes and highlight the importance of policy and practice reforms that are attuned to caregiver voices and lived realities.

The socio-demographic profile of the caregivers and children in this study provides critical insights into the lived experiences of families navigating the EHDI process in South Africa. The sample consisted exclusively of female caregivers, reinforcing the existing literature that highlights the predominant role of mothers in managing childhood hearing loss [29]. While this finding is consistent with global trends, studies in HICs indicate that fathers often play a more active role in intervention processes [30], suggesting a need for targeted awareness programmes to enhance paternal involvement in the South African context. The cultural expectations and social roles within the South African context may contribute to the lack of paternal involvement [31], necessitating gender-sensitive interventions to increase male participation in the EHDI process.

The caregivers in this study were ethnically diverse, though the majority were Black (5/9). This distribution reflects national demographics, where Black South Africans constitute the largest population group. However, based on published evidence, disparities in access to audiology services persist [12], with Black and lower-income caregivers often experiencing greater challenges in obtaining early diagnosis and intervention. This finding aligns with research in other LMICs, where systemic challenges such as healthcare inequities and financial constraints disproportionately affect marginalised groups [32]. This highlights the need for policies that specifically target equitable service delivery, particularly in under-resourced communities.

Educational attainment among caregivers varied, with two having Grade 11 or lower, three holding a matric certificate, and three possessing a post-matric diploma. Only one caregiver had a postgraduate degree. In this study, caregivers with higher levels of education and professional backgrounds were better equipped to understand their child’s condition, more likely to access EHDI services and navigate the EHDI system for their children, and advocate for timely support, highlighting a significant socio-educational disparity in service uptake. Participants with tertiary education demonstrated greater awareness of developmental milestones, stronger advocacy within the healthcare system, and a better understanding of available services. Their educational background appeared to influence their ability to recognise delays, seek appropriate referrals, and persist in follow-up, ultimately leading to improved access to services. These findings are consistent with previous research, which has shown that parental education levels are a critical social determinant of access to and engagement with early intervention services—influencing the timelines and quality of early intervention access [33]. Educational attainment can influence both health literacy and navigation of complex healthcare systems, further exacerbating inequities for those with limited formal education. Additionally, the education range in this study suggests that while some caregivers may have had access to higher education, others may have had limited exposure to information about paediatric hearing loss, potentially contributing to delayed intervention. Studies in LMICs have shown that caregiver education level is a key determinant of health literacy [33], influencing the ability to navigate complex healthcare systems effectively. Addressing health literacy through community-based education initiatives could help mitigate these challenges [34], ensuring that caregivers are well-informed about developmental milestones and available EHDI services.

### 4.1. Challenges to EHDI Access

Findings indicating systemic and structural challenges reveal delayed diagnosis and service fragmentation. The age of diagnosis for children in this study ranged from 4 to 30 months, with an average of 12 months, highlighting delays in identifying hearing loss. International best practices recommend newborn hearing screening before one month of age, diagnosis by three months, and early intervention by six months to optimise developmental outcomes [35]. In contrast, South Africa’s lack of UNHS leads to late identification, like patterns observed in other LMICs [36]. A critical issue is the lack of policy enforcement and integration of screening services into existing maternal and child healthcare programmes [36]. Service fragmentation was a recurring issue, with caregivers reporting multiple referrals before reaching an audiologist. This finding mirrors studies in sub-Saharan Africa, where a lack of streamlined referral systems contributes to diagnostic delays [37]. Fragmented services often require caregivers to navigate multiple facilities, increasing logistical burdens and prolonging the time before intervention is initiated. Strengthening inter-sectoral collaboration between primary healthcare providers and audiologists could facilitate a more efficient referral pathway and reduce unnecessary delays.

As far as logistical and financial challenges were concerned, barriers to accessibility of services emerged as a major challenge, with caregivers frequently travelling long distances to reach audiologists and therapy centres. For instance, one caregiver reported travelling 350 km daily for therapy, a challenge echoed in other African studies where audiological services are concentrated in urban centres, leaving rural populations underserved [12]. In contrast, HICs benefit from well-established tele-audiology services that reduce geographic challenges—an area that South Africa could expand to improve service delivery [38]. Investing in tele-health solutions and mobile screening units could significantly enhance accessibility in rural and remote areas. The high cost of amplification devices and related services posed significant challenges. Some caregivers struggled to secure funding for cochlear implants, hearing aids, and ongoing therapy, with medical aids not always covering the full expense. This finding aligns with research in LMICs where out-of-pocket expenses significantly impact intervention uptake [39,40]. Financial assistance, such as government subsidies and NGO-sponsored programmes, proved to be a crucial facilitator for lower-income families, emphasising the need for sustainable funding mechanisms. Expanding public–private partnerships could help bridge financial gaps, ensuring that cost is not a deterrent to early intervention.

When it comes to caregiver-related challenges limited awareness of hearing loss and milestones is an important finding [41,42,43]. Caregivers with lower educational backgrounds expressed difficulty recognising early signs of hearing loss, delaying intervention. In LMICs, inadequate public health campaigns on paediatric hearing loss contribute to low awareness levels, reinforcing the need for nationwide awareness initiatives that reach all educational and socio-economic groups. Increasing community-based outreach programmes that incorporate culturally relevant educational materials could improve caregiver recognition of early signs of hearing loss [44]. Szarkowski et al. [45] emphasise the importance of cultural sensitivity in FCEI programmes. These authors highlight that decision-making processes vary across cultures, with some families preferring collective decision-making involving extended family or community leaders. In the South African context, understanding these cultural dynamics is crucial for tailoring EHDI services that respect and align with familial and societal norms. Additionally, limited paternal involvement is a recurring theme in most caregiver related studies in the African context. Two caregivers reported that fathers played a minimal role in decision-making, particularly regarding cochlear implantation and therapy adherence. This aligns with findings in other African studies where gender norms influence caregiving roles, suggesting a need for targeted interventions to increase paternal participation in the EHDI process. Engaging fathers through male-focused education campaigns and family-centred counselling services could improve their involvement in decision-making processes.

### 4.2. Facilitators of EHDI Access

Caregiver knowledge and advocacy, family support and sign language training, and quality of healthcare and educational services emerged as important facilitators of EHDI access within the South African context. Firstly, caregivers who gained knowledge through experience became proactive advocates, not only for their children but also within their communities. One caregiver facilitated early diagnosis for another child in her community, demonstrating the potential of peer-driven awareness initiatives. This finding raises the importance of empowering caregivers with accurate information, enabling them to serve as agents of change in areas with limited audiology services. Creating formalised caregiver training programmes to equip parents with advocacy skills could strengthen community-led interventions. Secondly, counselling services and SASL training played a transformative role for caregivers. Parents who attended SASL training sessions reported improved communication with their children, enhancing their ability to support their child’s education. These findings align with international best practices that emphasise family-centred intervention approaches [46,47,48] and also align with foundational principles for FCEI-DHH, including early, timely, and equitable provision of support, and the importance of building partnerships with families [49]. Applying these principles can address systemic challenges identified in the study, such as delayed diagnoses and fragmented service delivery, by fostering collaborative relationships between caregivers and service providers. Mandating SASL training for caregivers of children who are DHH as part of EHDI services could improve long-term outcomes. Lastly, caregivers highlighted the positive impact of compassionate audiologists and specialised schools for DHH learners. Audiologists who provided both technical expertise and emotional support were valued highly, reinforcing the importance of clinician–patient relationships in long-term intervention success. Ensuring audiology training programmes incorporate holistic, patient-centred communication skills could further improve provider–patient relationships.

While this study provides valuable insights into the challenges and facilitators of EHDI in South Africa, certain limitations must be acknowledged. First, the study relied on a small, purposively selected sample of caregivers from a specific region, which may limit the generalizability of findings to broader populations. With nine caregivers participating, the findings may not capture the full range of experiences and challenges faced by all families navigating EHDI processes in South Africa. This sample, while diverse in ethnicity and education levels, may not adequately represent families from all socio-economic or geographical backgrounds, nor does it reflect the experiences of fathers or other caregiving figures. As a result, the transferability of the findings is limited and should be considered when interpreting the broader implications. Nonetheless, the rich, in-depth data provided by participants offers valuable contextual insights into systemic barriers, caregiver advocacy, and lived realities within a resource-constrained health system. While the sample size restricts generalisability in the quantitative sense, the depth and nuance of the qualitative accounts contribute meaningfully to understanding critical gaps and opportunities in EHDI in LMICs like South Africa. Future research should aim to include a larger and more varied cohort, including caregivers from underrepresented provinces and more male caregivers, to broaden the understanding of these issues. Additionally, the retrospective nature of the interviews may have introduced recall bias, as participants reflected on past experiences with EHDI services. Another limitation of the study linked to this is the inclusion of participants whose children accessed EI services as far back as 10 years ago. However, most of these children only entered intervention at significantly delayed ages—in some cases, not until they were six years old—due to systemic failures in early identification and service provision. While this introduces a temporal spread in experiences, South Africa’s EHDI landscape has not experienced major national reforms or universal implementation over the past decade. Therefore, the findings are still considered reflective of the current context. Nevertheless, caution is advised when generalising across all current provinces or populations. Language barriers may also have influenced data collection, despite efforts to conduct interviews in caregivers’ preferred languages. Furthermore, the study did not include perspectives from healthcare providers, which could have provided a more comprehensive understanding of systemic challenges. Future research should incorporate larger, more diverse samples and include the viewpoints of audiologists, speech therapists, and policymakers to develop a holistic approach to improving EHDI services in South Africa.

## 5. Conclusions

This study provides crucial insights into the challenges and facilitators influencing EHDI in South Africa. The findings highlight systemic, logistical, and caregiver-related challenges that hinder timely identification and intervention for children who are DHH. These challenges include delayed diagnosis due to healthcare provider practices, fragmented service delivery, financial constraints, and limited access to specialised audiology services. Additionally, the role of caregiver knowledge, family support services, and quality healthcare and educational resources emerged as key facilitators in navigating the EHDI process. Despite these challenges, caregivers demonstrated resilience and advocacy, actively seeking solutions to ensure their children’s access to hearing healthcare. Support services, including counselling, SASL training and financial aid, played a transformative role in enabling positive intervention outcomes. These findings underscore the urgent need for policy-driven improvements to enhance access, affordability and the efficiency of EHDI services.

To improve EHDI implementation in South Africa, several strategies must be prioritised, over and above capacity building for healthcare workers where comprehensive training programmes for HCWs must emphasise early identification of paediatric hearing loss, culturally congruent care, and the integration of caregivers as active participants in intervention planning. These strategies include (1) the scaling up and expansion of newborn hearing screening programmes where a national screening programme could significantly reduce the age of diagnosis and intervention; (2) greater investment in expanding tele-audiology services by leveraging digital health technologies to mitigate geographic challenges to care, especially in rural and remote regions; (3) the development and enhancement of sustainable financial support mechanisms where subsidised amplification devices, free therapy programmes, and increased medical aid coverage are put in place to ensure equitable access; (4) promotion of caregiver and paternal involvement and support where tailored education programmes for parents, including fathers, can improve adherence to intervention strategies and strengthen family support systems—with culturally and linguistically responsive service delivery that will strengthen family-centred intervention approaches; and (5) targeted awareness campaigns to enhance caregiver knowledge where national health initiatives should focus on educating caregivers about early hearing loss indicators and the importance of early intervention.

By addressing these challenges and building upon identified facilitators, South Africa can move toward achieving global EHDI benchmarks. Strengthening collaboration among policymakers, healthcare professionals, and community stakeholders is essential for creating an equitable and effective hearing healthcare system that ensures optimal outcomes for children who are DHH.

## Figures and Tables

**Table 1:** Participants’ socio-demographic profile.

Heading	Sub-Heading	Caregivers (N)	Children (N)
Gender	Male	0	5
	Female	9	4
Age	25–30	1	N/A
	31–36	3	N/A
	37–42	3	N/A
	43–48	2	N/A
Ethnicity	Black	5	9
	Multiracial	1	0
	Indian	1	2
	White	2	2
Marital Status	Married	6	N/A
	Single	3	N/A
Education Level	Grade 11 or lower	2	N/A
	Grade 12	3	N/A
	Post-Matric Diploma	3	N/A
	Baccalaureate Degree(s)	0	N/A
	Post Graduate Degree(s)	1	N/A
Employment	Employed	5	N/A
	Self-employed	2	N/A
	Student	0	N/A
	Unemployed	2	N/A
Home Language	English and SASL	N/A	1
	Sesotho	N/A	1
	Setswana	N/A	3
	Sepedi	N/A	2
	English	N/A	2
	Portuguese	N/A	1
	Afrikaans	N/A	1

Source: Original.

**Table 2:** Characteristics of children with hearing loss.

Child	Age When Hearing Loss Diagnosed (Months)	Age When Fitted with Amplification (Months)	Type of Amplification Device(s)
Thabo *	18	19	Hearing aid and cochlear implant
Lerato *	24	26	Hearing aid and cochlear implant
Karabo *	4	5	Hearing aid and cochlear implant
Dimpho *	20	24	Hearing aid and cochlear implant
Sizwe *	18	18	Bilateral hearing aids
Andrew *	27	27	Bilateral hearing aids
Paige *	6	6	Bilateral hearing aids
Josh *	22	23	Bilateral cochlear implants
Boikanyo *	14	20	Bilateral cochlear implants
Zack *	24	26	Bilateral cochlear implants
Mandla *	30	36	Bilateral cochlear implants

* These are not actual names of participants but their chosen pseudonyms. Source: Original.

## Data Availability

The data sets used and/or analysed during the current study are available from the corresponding author on a reasonable request.

## References

[1] Haile L.M., Kamenov K., Briant P.S., Orji A.U., Steinmetz J.D., Abdoli A., Abdollahi M. (2021). Hearing loss prevalence and years lived with disability, 1990–2019: Findings from the Global Burden of Disease study 2019. Lancet.

[2] Khoza-Shangase K. (2019). Early hearing detection and intervention in South Africa: Exploring factors compromising service delivery as expressed by caregivers. Int. J. Pediatr. Otorhinolaryngol..

[3] Manyisa N., Adadey S.M., Wonkam-Tingang E., Yalcouye A., Wonkam A. (2022). Hearing impairment in South Africa and the lessons learned for planetary health genomics: A systematic review. J. Integr. Biol..

[4] Wilson B.S., Tucci D.L., Merson M.H., O’Donoghue G.M. (2017). Global hearing health care: New findings and perspectives. Lancet.

[5] Bezuidenhout J.K., Khoza-Shangase K., De Maayer T., Strehlau R. (2018). Universal newborn hearing screening in public healthcare in South Africa: Challenges to implementation. S. Afr. J. Child Health.

[6] Bezuidenhout J.K. (2016). Feasibility Assessment of a Universal Newborn Hearing Screening Programme at Rahima Moosa Mother and Child Hospital. Master’s Thesis.

[7] Gina A., Bednarczuk N.F., Jayawardena A., Rea P., Arshad Q., Saman Y. (2021). Universal newborn hearing screening in South Africa: A single-centre study. BMJ Paediatr. Open.

[8] Maluleke N.P., Khoza-Shangase K., Kanji A. (2018). Hearing impairment detection and intervention in children from centre-based early intervention programmes. J. Child Health Care.

[9] Naidoo N. (2020). Early Hearing Detection and Intervention in KwaZulu-Natal: Analysis of Barriers and Facilitators from Guideline Generation to Clinical Application. Ph.D. Thesis.

[10] Scheepers L.J., Swanepoel D.W., le Roux T. (2014). Why parents refuse newborn hearing screening and default on follow-up rescreening-A South African perspective. Int. J. Pediatr. Otorhinolaryngol..

[11] Khan N.B., Joseph L., Adhikari M. (2018). The hearing screening experiences and practices of primary health care nurses: Indications for referral based on high-risk factors and community views about hearing loss. Afr. J. Prim. Health Care Fam. Med..

[12] Pillay M., Tiwari R., Kathard H., Chikte U. (2020). Sustainable workforce: South African Audiologists and Speech therapists. Hum. Resour. Health.

[13] Shung-King M., Orgill M., Slemming W. (2013). School health in South Africa: Reflections on the past and prospects for the future. S. Afr. Health Rev..

[14] Kanji A. (2018). Early hearing detection and intervention: Reflections from the South African context. S. Afr. J. Commun. Disord..

[15] Adugna M.B., Nabbouh F., Shehata S., Ghahari S. (2020). Barriers and facilitators to healthcare access for children with disabilities in low and middle income sub-Saharan African countries: A scoping review. BMC Health Serv. Res..

[16] Alduhaim A., Purcell A., Cumming S., Doble M. (2020). Parents’ views about factors facilitating their involvement in the oral early intervention services provided for their children with hearing loss in Kuwait. Int. J. Pediatr. Otorhinolaryngol..

[17] Keerthi S., Ramkumar V., Kumar S., Anand S. (2023). An exploratory study of nurses’ knowledge, skill, and training requirements for newborn hearing screening in a public-sector program in South India. J. Hear. Sci..

[18] Naidoo N., Khan N.B. (2022). Analysis of barriers and facilitators early hearing detection and intervention in Kwazulu-Natal. S. Afr. J. Commun. Disord..

[19] Health Professions Council of South Africa (2018). Early Hearing Detection and Intervention (EHDI) Guidelines. Health Professions’ Council of South Africa, Speech Language and Hearing Profess Board. https://www.hpcsa.co.za/Uploads/SLH/Guidelines%20for%20Early_Hearing_Detection_and_Intervention_(EHDI)_2018.pdf.

[20] Clandinin D.J. (2006). Narrative inquiry: A methodology for studying lived experience. Res. Stud. Music. Educ..

[21] Cresswell J.W., Plano Clark V.L. (2017). Designing and Conducting Mixed Methods Research.

[22] National Department of Health (2020). COVID-19 Disease: Infection Prevention and Control Guidelines.

[23] World Health Organization (2020). Coronavirus Disease (COVID-19). https://apps.who.int/iris/bitstream/handle/10665/336034/nCoV-weekly-sitrep11Oct20-eng.pdf.

[24] Braun V., Clarke V. (2013). Successful Qualitative Research: A practical Guide for Beginners.

[25] Braun V., Clarke V. (2020). One size fits all? What counts as quality practice in (reflexive) thematic analysis?. Qual. Res. Psychol..

[26] Vaismoradi M., Jones J., Turunen H., Snelgrove S. (2016). Theme development in qualitative content analysis and thematic analysis. J. Nurs. Educ. Pract..

[27] Braun V., Clarke V. (2019). Reflecting on reflexive thematic analysis. Qual. Res. Sport Exerc. Health.

[28] Cypress B.S. (2017). Rigor or reliability and validity in qualitative research: Perspectives, strategies, reconceptualization, and recommendations. Dimens. Crit. Care Nurs..

[29] Safari Z., Khoramshahi H., Nejad S.B., Dastoorpoor M., Moradi N. (2022). Coping and emotional styles in mothers of children with hearing impairment. J. Mod. Rehabil..

[30] Garcia I.L., Fernald L.C., Aboud F.E., Otieno R., Alu E., Luoto J.E. (2022). Father involvement and early child development in a low-resource setting. Soc. Sci. Med..

[31] Patel L., Mavungu E.M. (2016). ‘Children, families and the conundrum about men’: Exploring factors contributing to father absence in South Africa and its implications for social and care policies. S. Afr. Rev. Sociol..

[32] van Hees S.G., O’Fallon T., Hofker M., Dekker M., Polack S., Banks L.M., Spaan E.J. (2019). Leaving no one behind? Social inclusion of health insurance in low-and middle-income countries: A systematic review. Int. J. Equity Health.

[33] Hosseinpoor A.R., Bergen N., Chatterji S. (2013). Socio-demographic determinants of caregiving in older adults of low-and middle-income countries. Age Ageing.

[34] Nutbeam D. (2000). Health literacy as a public health goal: A challenge for contemporary health education and communication strategies into the 21st century. Health Promot. Int..

[35] Hyde M.L. (2005). Newborn hearing screening programs: Overview. J. Otolaryngol..

[36] Petrocchi-Bartal L., Khoza-Shangase K., Kanji A. (2025). Early intervention for hearing-impaired children—From policy to practice: An integrative review. Audiol. Res..

[37] Kanji A., Noorbhai W. (2021). Caregiver navigation through early hearing detection and intervention programs in South Africa: Caregiver navigation through EHDI. Infants Young Child..

[38] McMahon C.M. (2021). Guest editorial: Hearing care for all—An opportunity to globally unite to address inequities in hearing health. Ear Hear..

[39] Rahman T., Gasbarro D., Alam K. (2022). Financial risk protection from out-of-pocket health spending in low-and middle-income countries: A scoping review of the literature. Health Res. Policy Syst..

[40] Waterworth C.J., Marella M., O’Donovan J., Bright T., Dowell R., Bhutta M.F. (2022). Barriers to access to ear and hearing care services in low-and middle-income countries: A scoping review. Glob. Public Health.

[41] Yoshita S., Ranjeet G., Nisha K.V. (2024). Knowledge and attitudes of parents and caregivers in New Delhi to childhood hearing loss and hearing services. Indian J. Otolaryngol. Head Neck Surg..

[42] Scarinci N., Erbasi E., Moore E., Ching T.Y., Marnane V. (2018). The parents’ perspective of the early diagnostic period of their child with hearing loss: Information and support. Int. J. Audiol..

[43] Khoza-Shangase K., Khoza-Shangase K. (2022). Early hearing detection and intervention: Considering the role of caregivers as key co-drivers within the African context. Preventive Audiology: An African Perspective.

[44] Sánchez D., Adamovich S., Ingram M., Harris F.P., De Zapien J., Sánchez A., Colina S., Marrone N. (2017). The potential in preparing community health workers to address hearing loss. J. Am. Acad. Audiol..

[45] Szarkowski A., Moeller M.P., Gale E., Smith T., Birdsey B.C., Moodie S.T., Carr G., Stredler-Brown A., Yoshinaga-Itano C., Holzinger D. (2024). Family-centered early intervention deaf/hard of hearing (FCEI-DHH): Cultural & Global Implications. J. Deaf. Stud. Deaf. Educ..

[46] Maluleke N.P., Khoza-Shangase K., Kanji A. (2024). EHDI services should be accessible! Caregivers’ expectations of EHDI services in South Africa. Speech Lang. Hear..

[47] Findlen U.M., Davenport C.A., Cadieux J., Gehred A., Holt R.F., Vaughn L.M., Houston D., Hunter L.L. (2023). Barriers to and facilitators of early hearing detection and intervention in the United States: A systematic review. Ear Hear..

[48] Reed J., Findlen U.M., Davenport C., Deconde Johnson C., Sentelik M., Spangler C., Steuerwald W., Houston D. (2023). Parent and provider perspectives on early intervention in Ohio: A community collaborative approach. J. Early Hear. Detect. Interv..

[49] Moeller M.P., Gale E., Szarkowski A., Smith T., Birdsey B.C., Moodie S.T., Carr G., Stredler-Brown A., Yoshinaga-Itano C., Holzinger D. (2024). Family-centered early intervention Deaf/Hard of Hearing (FCEI-DHH): Foundation principles. J. Deaf. Stud. Deaf. Educ..

